# Re-Bestrahlung und Bevacizumab – wirksame Partner bei rezidiviertem Glioblastom

**DOI:** 10.1007/s00066-023-02084-8

**Published:** 2023-04-20

**Authors:** Clemens Seidel, Nils H. Nicolay

**Affiliations:** Klinik und Poliklinik für Strahlentherapie, Universitätsklinikum Leipzig, Stephanstraße 9a, 04103 Leipzig, Deutschland

## Hintergrund

Im Rahmen der vorliegenden Studie sollte eingeschätzt werden, ob eine Re-Bestrahlung in Kombination mit Bevacizumab das Gesamtüberleben (OS) und/oder progressionsfreie Überleben (PFS) von Patienten mit Glioblastom-Rezidiv gegenüber Bevacizumab alleine verbessert. Der primäre Endpunkt der Studie war das OS, sekundäre Endpunkte umfassten das PFS sowie Ansprechraten und Toxizitäten der Therapie einschließlich nachhängender ZNS-Nebenwirkungen.

## Methoden

NRG Oncology/RTOG1205 war eine prospektive Phase-2-Studie zu Re-Bestrahlung und Bevacizumab versus Bevacizumab allein. Stratifizierende Faktoren umfassten Alter, Resektionsausmaß und den mittels Karnofsky Performance Status (KPS) quantifizierten Allgemeinzustand. Patienten mit rezidiviertem GBM und bildgebendem Nachweis einer Tumorprogression > 6 Monate nach Beendigung einer Radiochemotherapie wurden eingeschlossen. Die Patienten wurden 1:1 zwischen Bevacizumab-Monotherapie (10 mg/kg) alle zwei Wochen bis zur Tumorprogression oder Bevacizumab mit simultaner Re-Bestrahlung (35 Gy in 10 Fraktionen) randomisiert.

## Ergebnisse

Zwischen Dezember 2012 und April 2016 wurden 182 Patienten randomisiert, von den 170 die Einschlusskriterien erfüllten. Die Patientencharakteristika waren zwischen beiden Behandlungsarmen balanciert. Die Patienten wurden im Median für 12,8 Monate nachbeobachtet. Das Gesamtüberleben betrug 10,1 Monate im Kombinationsarm und 9,7 Monate im Monotherapiearm; damit konnte keine Verbesserung des Gesamtüberlebens durch die Kombination aus Bevacizumab und Re-Bestrahlung gezeigt werden (HR: 0,98, 80 % CI: 0,79–1,23, *p* = 0,46). Das mediane PFS für Bevacizumab und Re-Bestrahlung betrug 7,1 Monate vs. 3,8 Monate im Bevacizumab-Arm (HR: 0,73; 95 % CI, 0,53–1,0; *p* = 0,05) und das PFS nach 6 Monaten lag bei 54,3 % (95 % CI, 43,5–65,1) für Bevacizumab und Re-Bestrahlung (*p* = 0,001) gegenüber 29,1 % für die alleinige Behandlung mit Bevacizumab (95 % CI, 19,1–39,1). Insgesamt wurde die Rezidivtherapie gut toleriert. Die Rate von höhergradigen Toxizitäten (≥ Grad 3) nach Kombinationsbehandlung mit Re-Bestrahlung und Bevacizumab betrug 5 %; verzögerte hochgradige AE wurden nicht beschrieben. Die meisten Patienten verstarben am Glioblastom-Rezidiv.

## Schlussfolgerung

NRG Oncology/RTOG1205 ist die erste prospektive randomisierte multzentrische Studie zur Untersuchung der Sicherheit und Wirksamkeit der Re-Bestrahlung bei Patienten mit Glioblastom-Rezidiven unter Anwendung moderner Bestrahlungstechniken. Die Re-Bestrahlung erschien sicher und gut verträglich. Die Kombination aus Bevacizumab und RT zeigte einen relevanten Vorteil hinsichtlich des PFS, insbesondere nach 6 Monaten, ohne jedoch das Gesamtüberleben zu verbessern.

## Kommentar

In Zusammenschau mit älteren Daten [1, 2] verbessert die vorliegende Studie erheblich die Evidenz zur Re-Bestrahlung bei Glioblastom-Rezidiven. Zum ersten Mal werden Sicherheit und Verträglichkeit einer Re-Bestrahlung während einer begleitenden Bevacizumab-Therapie in einem kontrollierten Setting analysiert und gezeigt. Zudem konnte in der Studie durch die Kombination aus Re-Bestrahlung und Bevacizumab das progressionsfreie Überleben signifikant verlängert werden. In Anbetracht der nachgewiesenen abträglichen Effekte eines Tumorrezidivs auf Lebensqualität und neurologische Funktion, erscheint eine Verlängerung des PFS als relevanter Therapievorteil, der im ZNS deutlich schwerer wiegt als bei vielen Tumorerkrankungen außerhalb des ZNS [3, 4]. Wie bekannt, hat in den USA beispielsweise allein der positive PFS-Effekt von Bevacizumab [5, 6] ausgereicht, um das Medikament dort zur Therapie von Glioblastom-Rezidiven zuzulassen. Die Kombination von Re-Bestrahlung mit Bevacizumab erscheint auch unter Verträglichkeitsaspekten sinnvoll, da durch den Anti-VEGF Effekt auch das Risiko symptomatischer Pseudoprogressionen bzw. Nekrosen nach Re-Bestrahlung minimiert wird [7, 8].

In der Subgruppenanalyse der Studie schienen besonders Patienten in sehr gutem Zustand von der Kombinationstherapie zu profitieren; so bestand bei Patienten mit KPS 90–100 ein Trend für eine Verbesserung des Gesamtüberlebens (HR 0,67; 95 % CI, 0,40–1,13; *p* = 0,13). Wichtig für die Strahlentherapie ist natürlich auch, dass im Rahmen der Studie ein einheitliches Dosis- und Zielvolumenkonzept verwendet wurde, welches somit in seiner praktischen Anwendung für Tumoren < 6 cm nun als bester, evidenzbasierter Standard betrachtet werden kann. Als GTV waren die Resektionshöhle und eventuelle Kontrastmittel-anreichernde Resttumoranteile definiert sowie zusätzlich eine isotrope Expansion zum PTV von 3–5 mm. Bei kleinen Tumoren < 3,5 cm war die Definition eines zusätzlichen CTV mit einem GTV-CTV-Margin von max. 5 mm optional. Das erlaubte Dosismaximum (0,03 cm^3^) im PTV betrug 42,0 Gy. 85 % der Bestrahlungen erfolgten als intensitätsmodulierte Bestrahlung, 2 Patienten erhielten eine Protonentherapie.

Die im Rahmen der Re-Bestrahlung behandelten Volumina erscheinen mit im Mittel 19 cm^3^ (0,4–208 cm^3^) für das GTV und 53 cm^3^ (4–411 cm^3^) für das PTV relativ klein, auch wegen der engen Sicherheitssäume. Trotzdem zeigte eine im Rahmen der ASTRO-Konferenz 2019 vorgestellte Analyse der post-hoc erhobenen Planqualität, dass nur bei etwa 60 % der Patienten im Re-Bestrahlungs-Arm die in der Studie prädefinierten Planungsziele erreicht werden konnten [9]. Das für eine zerebrale Re-Bestrahlung häufig in der klinischen Praxis zugrunde gelegte Einschlusskriterium von mindestens 6 Monaten Abstand zur primären Bestrahlung wurde im Verlauf der Studie aufgrund von langsamer Rekrutierung aufgeweicht, so dass auch kürzere Intervalle zulässig waren. Aufgrund des Studiendesigns bleibt allerdings offen, ob eine hypofraktionierte Re-Bestrahlung mit 35 Gy – insbesondere in schneller Folge zur Primärtherapie – auch ohne Bevacizumab wirksam und verträglich angewandt werden kann. Normofraktionierte Konzept bei größeren Zielvolumina oder in der Nähe von Risikostrukturen erscheinen im Rahmen einer Re-Bestrahlung zur Vermeidung von Radionekrosen in der klinischen Praxis durchaus erwägenswert. Eine weitere wichtige Frage zur Planungsbildgebung für eine optimierte Zielvolumendefinition (MRT vs. Aminosäure-PET/CT), wird aktuell im Rahmen der GLIAA-Studie beforscht [10].

Nach Publikation der aktuellen Studie ist die Re-Bestrahlung der bisher einzige Kombinationspartner zu Bevacizumab, für den im Gegensatz zu medikamentösen Partnern wie z. B. Lomustin [11, 12] ein Vorteil für das Outcome nachgewiesen werden konnte. Auch im Hinblick auf die onkologischen Ergebnisse der üblichen Rezidiv-Chemotherapie mit Lomustin und einem medianen PFS von 1,5 Monaten schneidet das hier beschriebene Behandlungskonzept mit einem PFS von 7,1 Monaten sehr gut ab [13].

Anzumerken ist, dass zur Beurteilung der Tumorprogression sowohl die McDonald- als auch die RANO-Kriterien verwendet wurden, ohne dass zwischen beiden Beurteilungsschemata im Ergebnis signifikante Unterschiede bestanden.

Trotz aller vielversprechenden Ergebnisse weist die vorliegende Studie auch eindeutige Limitationen auf: Insbesondere fehlte ein zentrales radiologisches Review sowie ein zentrales prospektives Review der Bestrahlungspläne. Außerdem lag bei fast der Hälfte der in die Studie eingeschlossenen Patienten in beiden Behandlungsarmen keine Information zum Methylierungsstatus des MGMT-Promotors vor. Diese Information wäre aber zur Einordnung der Prognose der Patienten bzw. zur Beurteilung beider Therapiearme sehr wünschenswert gewesen. Im Rahmen der durch das Phase-2-Design bedingten moderaten Studiengröße und der letztlich sehr heterogenen Kohorte mit verschiedenen Rezidivsituationen erscheint das Erreichen des primären Endpunktes, nämlich eine Verbesserung des Gesamtüberlebens durch die Kombinationstherapie zu belegen, sehr ambitioniert.

## Fazit

Die Kombination aus Re-Bestrahlung und Bevacizumab erscheint auf der Basis der vorliegenden randomisierten Phase 2‑Studie als bisher wirksamste Therapie bei Glioblastom-Rezidiven. Aufgrund der fehlenden Verbesserung des Gesamtüberlebens als primärem Endpunkt ist die RTOG1205-Studie formal negativ. Trotzdem bietet das deutlich verlängerte PFS bei sehr guter Verträglichkeit eine starke Rationale, die Kombinationstherapie für einen potenziellen Einsatz in der klinischen Routineversorgung von Glioblastom-Rezidiven weiter zu untersuchen.

*Clemens Seidel und Nils H. Nicolay, Leipzig*
